# Anthocyanins as Immunomodulatory Dietary Supplements: A Nutraceutical Perspective and Micro-/Nano-Strategies for Enhanced Bioavailability

**DOI:** 10.3390/nu15194152

**Published:** 2023-09-26

**Authors:** Thadiyan Parambil Ijinu, Lorenza Francesca De Lellis, Santny Shanmugarama, Rosa Pérez-Gregorio, Parameswaran Sasikumar, Hammad Ullah, Daniele Giuseppe Buccato, Alessandro Di Minno, Alessandra Baldi, Maria Daglia

**Affiliations:** 1 Naturæ Scientific, Kerala University-Business Innovation and Incubation Centre, Kariavattom Campus, University of Kerala, Thiruvananthapuram 695581, India; ijinutp@gmail.com; 2 The National Society of Ethnopharmacology, VRA-179, Mannamoola, Peroorkada P.O., Thiruvananthapuram 695005, India; 3 Department of Pharmacy, University of Napoli Federico II, Via D. Montesano 49, 80131 Naples, Italy; lo.delellis2@libero.it (L.F.D.L.); d.buccato@studenti.unina.it (D.G.B.); alessandro.diminno@unina.it (A.D.M.); alessandra.baldi.alimenti@gmail.com (A.B.); 4 Department of Pathology, The University of Oklahoma Health Sciences Center, Oklahoma City, OK 73104, USA; santny.biotech@gmail.com; 5 Food and Health Omics Group, Institute of Agroecology and Food, Faculty of Sciences, University of Vigo, 32004 Ourense, Spain; maria.gregorio@fc.up.pt; 6 LAQV-REQUIMTE, Department of Chemistry and Biochemistry, Faculty of Sciences, University of Porto, 4169-007 Porto, Portugal; 7 Department of Analytical and Food Chemistry, Galicia Sur Health Research Institute (IISGS), SERGAS-UVIGO, 32002 Ourense, Spain; 8 Drug Testing Laboratory, Government Ayurveda College, Thiruvananthapuram 695001, India; sashparameswaran@gmail.com; 9 CEINGE-Biotecnologie Avanzate, Via Gaetano Salvatore 486, 80145 Naples, Italy; 10 International Research Center for Food Nutrition and Safety, Jiangsu University, Zhenjiang 212013, China

**Keywords:** anthocyanins, immune cells, gut microbiota, bioavailability, efficacy, nanodelivery methods

## Abstract

Anthocyanins (ACNs) have attracted considerable attention for their potential to modulate the immune system. Research has revealed their antioxidant and anti-inflammatory properties, which play a crucial role in immune regulation by influencing key immune cells, such as lymphocytes, macrophages, and dendritic cells. Moreover, ACNs contribute towards maintaining a balance between proinflammatory and anti-inflammatory cytokines, thus promoting immune health. Beyond their direct effects on immune cells, ACNs significantly impact gut health and the microbiota, essential factors in immune regulation. Emerging evidence suggests that they positively influence the composition of the gut microbiome, enhancing their immunomodulatory effects. Furthermore, these compounds synergize with other bioactive substances, such as vitamins and minerals, further enhancing their potential as immune-supporting dietary supplements. However, detailed clinical studies must fully validate these findings and determine safe dosages across varied populations. Incorporating these natural compounds into functional foods or supplements could revolutionize the management of immune-related conditions. Personalized nutrition and healthcare strategies may be developed to enhance overall well-being and immune resilience by fully understanding the mechanisms underlying the actions of their components. Recent advancements in delivery methods have focused on improving the bioavailability and effectiveness of ACNs, providing promising avenues for future applications.

## 1. Introduction

The immune system is vital in safeguarding the body against disease and maintaining its physiological functions. Any disturbance in the immune system can result in various health problems, such as autoimmune diseases, inflammatory disorders, cancer, etc. A debilitated immune system can lead to infections and tumors [1] while an overactive one may cause autoimmune conditions, such as type I diabetes, systemic lupus erythematosus, and rheumatoid arthritis [2,3]. Immune responses can be categorized into two types: innate and adaptive. Innate immunity comprises pre-existing responses triggered without prior exposure to an antigen and is inherent in individuals from birth [4]. Conversely, adaptive immunity includes humoral and cell-mediated immunity, activated only after exposure to a specific antigen [5]. Immune cells, such as lymphocytes, dendritic cells, monocytes/macrophages, natural killer cells, CD^4+^ and CD^8+^ T-cells, and myeloid-derived suppressor cells, play crucial roles in regulating innate and adaptive immunity through their distinct structures and functions [6]. In addition to these immune cells, various inflammatory cytokines and chemokines, including interleukins (ILs), tumor necrosis factor-alpha (TNF-α), transforming growth factor beta (TGF-β), and interferon-gamma (IFN-γ), are triggered and regulated [7,8,9]. Signaling pathways, such as nuclear factor kappa B (NF-κB), programmed cell death protein 1 (PD-1), cytotoxic T-lymphocyte–associated antigen 4 (CTLA-4), mitogen-activated protein kinases (MAPKs), extracellular signal-regulated kinase (ERK), c-Jun *N*-terminal kinase (JNK), Janus kinase/signal transducer and activator of transcription (JAK/STAT); nucleotide binding; and oligomerization domain (NOD)-like receptors (NLRs) also participate in immunomodulation [10,11,12]. Modifying the immune system involves enhancing, inhibiting, activating, amplifying, or expressing various elements or processes within the normal immune response. This modulation ultimately leads to an improvement in responsiveness to pathogens.

Amidst the global COVID-19 or coronavirus pandemic, it is evident that markets worldwide have become inundated with a plethora of products claiming to be “immunity boosters”. These offerings emerge alongside various speculative cures, treatments, and preventive strategies. However, it is crucial to recognize that the notion of an “immunity booster” is scientifically misleading and frequently exploited to promote unverified products and therapies [13,14]. The “immunity booster” market comprises vitamins, minerals, antioxidants, probiotics, functional foods, nutraceuticals, and other complementary and alternative medicines. In a study conducted using data from a US National Health and Nutrition Examination Survey, more than 50% of the US population acknowledged their use of supplements [15]. This widespread usage has significant economic implications, with the global dietary supplement market estimated to be approximately USD 133.1 billion, projected to accelerate at a CAGR of 9.6% from 2016 to 2024 [16]. It is crucial to underscore that promoting “immunity boosters” can mislead consumers and give rise to false hopes. The scientific community consistently emphasizes the importance of evidence-based practices and rigorous research to substantiate the claims of any product or therapy. Unfortunately, many of the offerings currently flooding the market lack the necessary scientific backing to validate their effectiveness.

Natural products have been extensively studied for their potential immunomodulatory properties [17]. These natural products can modulate the immune system by enhancing or suppressing the immune response. They can also promote the production of cytokines and other immune system molecules, leading to an overall improvement in immune function. One of their key functions is to stimulate non-specific innate immune responses, which involve the use of immune system mediators, such as innate leukocytes (natural killer cells, eosinophils, basophils, and mast cells) and phagocytic cells (neutrophils, macrophages, and dendritic cells), to defend against pathogens [18]. By enhancing innate immunity, these products facilitate effector innate immune responses, such as immune cell infiltration, phagocytosis, and cytotoxic mechanisms, such as natural-killer-cell-mediated cytotoxicity, ultimately destroying tumor cells [19]. Certain natural compounds also act as immunomodulatory agents, activating the adaptive immune system. In adaptive immunity, T- and B-lymphocytes recognize tumor antigens through cell-surface antigen-specific receptors, leading to an augmented humoral response and the elimination of tumor cells through various mechanisms, including T-cell mediated cancer cell death [20].

Insufficient nutrition, malnutrition, or deficiencies in specific nutrients can disrupt the functioning of the immune system, leaving the body vulnerable to disease. To maintain a robust immune system, it is necessary to consume a well-balanced diet that provides all of the necessary nutrients in appropriate quantities [21]. Functional foods and nutraceuticals offer an alternative approach to boosting immune function and support the management of diverse diseases. Several studies have explored the potential of plant-based nutraceuticals as immunomodulating agents, owing to their wide range of effects that can positively impact the immune system [22,23,24]. These substances are often better tolerated than conventional pharmaceutical treatments, making them an attractive supplement for enhancing immune system function [21]. Among them, ACNs, naturally occurring water-soluble flavonoids, have been shown to stimulate immunomodulatory and antioxidant effects. This, in turn, helps to reduce the harmful cooperative and synergistic effects of oxidative stress and proinflammatory cytokines. Consequently, ACN may protect against chronic diseases [25,26]. This review provides a comprehensive update on the current scientific understanding of the basis underlying immunomodulatory properties, and the clinical significance of ACNs, thereby highlighting their potential as dietary supplements/nutraceuticals in the rapidly expanding global food industry.

## 2. Chemistry and Natural Sources of ACNs

**Figure 1:**
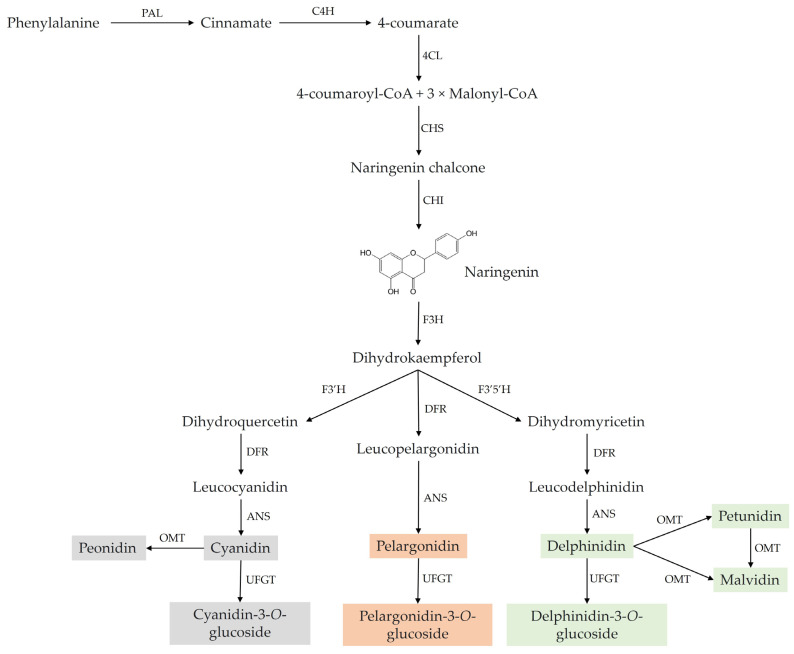
Overview of the phenylpropanoid pathway. PAL, phenylalanine ammonia-lyase; C4H, cinnamic acid 4-hydroxylase; 4CL, 4-coumarate:CoA ligase; CHS, chalcone synthase; CHI, chalcone isomerase; F3H, flavanone3-hydroxylase; F3′H, flavonoid 3′-hydroxylase; F3′5′H, flavonoid 3′5′-hydroxylase; DFR, dihydroflavonol 4-reductase; ANS, anthocyanidin synthase; OMT, ortho-methyltransferase; UFGT, UDP flavonoid glycosyltransferase.

ACNs are the glucosides of anthocyanidins and are predominantly found as 3-glucosides or acylglycosides. The primary configuration of their parent nucleus consists of a strongly conjugated 2-phenyl-benzopyran cation. Three carbon atoms connect the benzene rings to form a C6–C3–C6 skeleton, the common motif of ACNs [27,28]. These ACNs can be classified into sugar-free anthocyanidin aglycones and glycosides. ACNs include over 700 different derivatives of 27 aglycons [29]. These compounds are formed, via the phenylpropanoid pathway (Figure 1), from anthocyanidins, which serve as their precursors [30].

**Figure 2:**
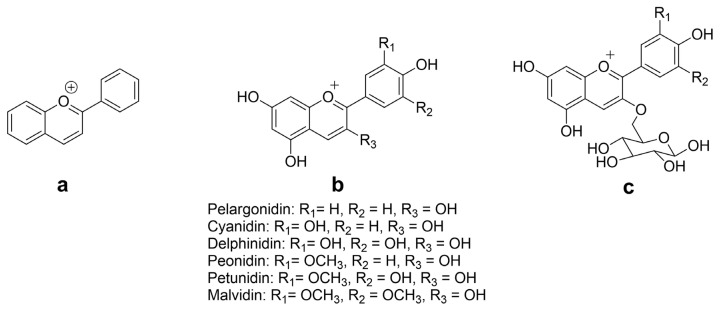
Chemical skeleton of anthocyanin; (**a**) flavylium cation, (**b**) most common anthocyanidins, (**c**) basic structure of anthocyanin.

The chemical structures of the flavylium cation and ACNs are given in Figure 2. The aglycone forms of anthocyanidins are rarely observed due to their inherent instability and reactivity, primarily caused by the electron-deficient flavylium cation. ACNs are more commonly observed in a glycosylated state as this modification enhances their stability and solubility [31]. They are typically composed of one of six anthocyanidin bases, which differ in their molecular structure at the B-ring and are attached to a sugar moiety at the third position of the C-ring. Approximately 90% of all known anthocyanidins found in plants consist of six bases, namely, pelargonidin, cyanidin, peonidin, delphinidin, petunidin, and malvidin [32,33], with the glycosides of the nonmethylated anthocyanidins (cyanidin, delphinidin, and pelargonidin) being the most abundant natural ACNs, accounting for up to 60% of the total content [34].

The color of the ACNs becomes bluer as the number of hydroxyl groups in the B-ring increases—conversely, methylation results in a red shift in the color of the ACNs. Moreover, the color is also pH dependent, i.e., blue in basic conditions and red in acidic conditions (where ACNs are positively charged). Methylation of the B-ring offers improved resistance to oxidation and helps stabilize the ACNs. Methyl-modified flavonoids are commonly present on the surfaces of leaves and flowers [35]. On the other hand, the glycosylation of ACNs causes a hypsochromic shift in the absorption maxima of the spectra and enhances their stability during their storage in vacuoles [36,37]. Sugar molecules, such as glucose, galactose, rhamnose, or arabinose, are primarily attached to the aglycone to form derivatives of 3-glycosides or 3,5-diglycosides [25]. The sugar moieties of acylated ACNs, usually attached to the hydroxyl group in C-3 and C-5 of the aglycone, have a covalent ester linkage to one or more of the aliphatic (acetic, malonic, oxalic, and succinic) or aromatic (caffeic, coumaric, ferulic, hydroxybenzoic, and sinapic) acids [38,39]. The acylation of glycosyl moieties in ACNs alters their chemical properties, enhancing stability. Aliphatic acylation does not affect color while aromatic acylation causes a blue shift. As a result, acylated ACNs are more suitable for use as natural colorants and bioactive components in innovative functional foods and nutraceuticals. This modification improves stability and expands their potential applications [37,39,40,41].

Many fruits are rich in ACNs, which serve as the pigments responsible for their vibrant colors. Berries, like blackcurrants, blackberries, blueberries, and cranberries, and vegetables, like black carrots, red cabbage, and purple potato, are well-known sources of ACNs [42,43]. For instance, pistachios contain significant amounts of cyanidin 3-*O*-galactoside while blackcurrants contain delphinidin 3-*O*-rutinoside and cyanidin 3-*O*-rutinoside. Red wine, elderberries, and pomegranate juice are known to contain malvidin 3-*O*-glucoside, cyanidin 3-*O*-glucoside, and cyanidin 3,5-*O*-diglucoside, respectively [44,45]. These compounds exhibit strong antioxidant properties and have the potential to function as preventive bioactive molecules against various illnesses. Additionally, various flowers, particularly those with red, purple, and blue shades, are used in traditional medicine or are consumed as food. These flowers, such as red clover, red rose, red hibiscus, red pineapple sage, pink blossom, blue rosemary, blue chicory, cornflower, purple passion flower, purple mint, common violet, purple sage, and lavender, are also rich in ACNs [34]. Acylated ACNs with varying structures are also present in fruits, berries, vegetables, and tubers. Some rich sources of these compounds include purple sweet potato, red radish, purple carrot, and red cabbage [46]. Within the pigmented members of the Solanaceae family, such as potatoes, peppers, tomatoes, and eggplants, acylated ACNs are identified by the structure of anthocyanidin-3-hydroxycinnamoyl-rutinoside-5-glucoside, with delphinidin being the primary anthocyanidin, excluding pigmented potatoes [39,47].

## 3. Immunomodulatory Potential of ACNs

Over the years, researchers have explored various avenues to improve immune function and address immune-related disorders. Recently, there has been growing interest in the potential of natural compounds to influence the immune response. ACNs, documented in both in vitro and in vivo studies, have been found to possess diverse health-promoting properties. In addition to their notable antioxidant property, ACNs play a crucial role in modulating the immune system. They protect the immune system through the following cellular processes:

- ACNs exhibit strong antioxidant characteristics, effectively neutralizing free radicals and diminishing oxidative stress. By safeguarding immune cells against oxidative damage, these compounds preserve the integrity and functionality of the immune system [21,33,48,49];
- Inflammation disrupts immune homeostasis, leading to diverse diseases or disease conditions. However, ACNs have been proven to play a crucial role in regulating inflammatory pathways by inhibiting the production of proinflammatory cytokines (IL-1β, IL-6, and TNF-α) and proinflammatory mediators (COX, LOX, MPO, and PGE_2_), thereby offering protection against the development of various inflammatory conditions [29,32,50];
- ACNs influence immune cell activation and proliferation by modulating various pathways. Moreover, they have a significant impact on gene expression within immune cells, leading to the heightened expression of genes responsible for antioxidant defense, anti-inflammatory pathways, and immune cell activation [29,51,52];
- Recent research indicates that ACNs can influence gut microbiota composition and functionality. Given the pivotal role of the gut microbiota in regulating the immune system, this interaction has the potential to contribute significantly to the immunomodulatory effects exhibited by ACNs [53,54,55,56,57].

ACNs, along with other polyphenols, such as flavones, flavone-3-ols, and flavanones, have shown potential in promoting a balanced T helper cell type 1 (Th1)/Th2 response and reducing the production of allergen-specific immunoglobulin (Ig) E antibodies [58]. Additionally, ACNs can activate gamma-delta (γδ) T cells, a type of immune cell involved in both acquired and innate immunity, by mimicking pathogen-associated molecular patterns [59]. This interaction with γδ T cells enhances the activity of essential immune components, such as natural killer cells, cytokines, and lymphocytes, which are crucial in defending against pathogens that can enter the body through the digestive and respiratory systems. Cyanidin 3-*O*-glucoside inhibits osteoclast differentiation and formation in a dose-dependent manner by downregulating osteoclast differentiation marker genes and suppressing the activation of specific kinases. It specifically targets osteoclasts without affecting osteoblasts and promotes osteoblast differentiation and matrix formation [60]. Protocatechuic acid (PCA), a major metabolite of ACNs, dose-dependently inhibited the differentiation of osteoclasts and suppressed their bone-resorbing activity by targeting the JNK signaling pathway and down-regulating osteoclastogenesis-related genes. Moreover, the administration of PCA effectively restored bone loss in mice induced by lipopolysaccharide, suggesting a potential therapeutic application for inflammatory bone disorders [61].

Delphinidin increased cytosolic-free Ca^2+^ concentration by releasing Ca^2+^ from intracellular stores and enhancing Ca^2+^ entry through the putative Ca^2+^ release-activated Ca^2+^ (CRAC) channel. This led to the activation of the nuclear factor of activated T cells (NFAT) and subsequent stimulation of cytokine production, particularly IL-2 and IFN-γ, indicating its immunostimulatory effects on T cells through the CRAC channel and NFAT pathway [62]. These inhibitory actions are associated with the ortho-dihydroxy phenyl structure on the B-ring of anthocyanidins. Anthocyanidins with an ortho-dihydroxy phenyl structure on the B-ring exhibited inhibitory effects on cell transformation and activator protein-1 transactivation induced by 12-*O*-tetradecanoylphorbol-13-acetate (TPA). Delphinidin, but not peonidin, specifically blocked the phosphorylation of protein kinases in the ERK and JNK pathways while p38 kinase was unaffected. Moreover, specific inhibitors of JNK and ERK, but not p38, were found to block cell transformation, indicating that anthocyanidins inhibit tumorigenesis by targeting the MAPK pathway [63]. Delphinidin, the most potent inhibitor, suppressed cyclooxygenase (COX)-2 expression by blocking MAPK-mediated pathways, including the activation of NF-κB, activator protein-1 (AP-1), and CCAAT/enhancer binding protein-delta (C/EBPδ). This provides molecular evidence for the anti-inflammatory properties of anthocyanidins with an ortho-dihydroxyphenyl structure by inhibiting MAPK-mediated COX-2 expression [64].

In a recent study, researchers investigated the immune-modulatory effects of *Sambucus ebulus* L. fruit infusion, which contains significant amounts of cyanidin 3-*O*-galactoside (Cy3Gal, 48.15 mg/g dry weight) and cyanidin 3-sambubioside (43.41 mg/g dry weight). The study involved healthy volunteers, with fifty-three participants under a 4-week intervention. The intervention results were promising, as a noteworthy reduction in various markers of inflammation and complementary activity was observed. Specifically, levels of total protein decreased by 2.82%, IL-6 by 20.15%, TNFα by 5.38%, IL-8 by 5.50%, C3 by 4.16%, and C4 by 14.29%. Interestingly, hemoglobin and hematocrit levels decreased across the entire group, particularly among women. This indicates that consuming *S. ebulus* fruits could enhance the functioning of the immune system [65]. Another study investigated the anti-inflammatory properties of a specific fraction of black rice, rich in cyanidin 3-*O*-glucoside and peonidin 3-*O*-glucoside. The researchers found that this fraction effectively suppressed the expression of inflammatory genes (NLRP3, IL-1β, and IL-18) and the secretion of cytokines (IL-6, IL-1β, and IL-18) induced by the spike glycoprotein S1 subunit of SARS-CoV-2 in A549 and THP-1 cells. Moreover, the black rice fraction inhibited the activation of NF-κB and downregulated inflammasome-dependent inflammatory pathway proteins (NLRP3, ASC, and caspase-1). These results indicate that ACNs from black rice could be utilized in preventive strategies against the long-term effects of COVID-19 infection [66].

Cyanidin 3-*O*-glucoside (C3G) was delivered using enteric sodium alginate to assess its anti-food allergy effects in vivo. The targeted rectal and colonic delivery of C3G proved highly effective in reducing allergic symptoms, diarrhea, and serological markers. Additionally, C3G improved the intestinal barrier, increased secretory IgA and β-defensin, and regulated the Th1/Th2 immune balance. Beneficial bacteria, including *Lactobacillus* and *Odoribacter*, thrived while pathogenic bacteria, such as *Helicobacter* and *Turicibacter*, decreased [67]. A study investigated ACN-rich roselle extract (ARRE) from *Hibiscus sabdariffa* in broiler chickens; diets supplemented with ARRE increased antioxidant capacity, lysozyme levels, antimicrobial enzymes, and anti-inflammatory cytokine IL-10. At 200 and 400 mg/kg body weight doses, the ARRE resulted in increased levels of serum complement 3, indicating an enhanced immune response [68]. Furthermore, consuming C3G provided lasting cardioprotection against ischemia/reperfusion injury, independent of its anti-inflammatory properties. Immune-deficient mice experienced similar protection, suggesting other mechanisms at play. The C3G-enriched diet altered the gut microbiome and transferring fecal microbiota from C3G-fed mice replicated the cardioprotective effects [69].

A research study explored the potential effects of C3G on Alzheimer’s disease (AD) and other conditions by analyzing gene expression patterns in the spleen. The investigation involved wild-type mice (C57BL/6J Jms), mice with an Alzheimer’s disease model (APPswe/PS1dE9 mice), and mice with the Alzheimer’s model treated with C3G. By comparing the gene expression profiles, the study identified specific genes related to antioxidants, immune responses, and AD pathways that were differentially expressed in the C3G-treated group. Notably, six crucial antioxidant genes (S100a8, S100a9, Prdx2, Hp, Mpst, and Prxl2a) and various immune-related genes were upregulated in the treated mice. These findings support the potential of C3G to have immunomodulatory effects [70]. Another investigation focused on the effects of a boysenberry and apple juice concentrate rich in cyanidin glycosides (including cyanidin 3-*O*-sophoroside, cyanidin 3-*O*-glucoside, and cyanidin 3-*O*-[2-glucosylrutinoside]) on acute lung inflammation and M2 macrophage-associated cytokines in a male C57BL/6J mouse model of allergic airways disease. The mice received oral administration of the concentrate (equivalent to 0.2 mg/kg in humans; 2.5 mg/kg total ACN content) prior to an intranasal ovalbumin challenge. Consumption of the concentrate led to significant reductions in eosinophil, neutrophil, and T-cell infiltration in the lung and mucous production. Furthermore, gene expression analysis revealed increased expression of anti-inflammatory macrophage markers (Arg1) and specific cytokines (CXCL10 and CCL4) associated with an anti-inflammatory response [71]. In SARS-CoV-2 viral replication, the papain-like (PLpro) and main proteases (Mpro) are crucial in processing viral replicase polypeptides. The PLpro protease also possesses deubiquitinating activity, impacting important signaling pathways, inhibiting the interferon response, and antagonizing innate immune responses. Interestingly, C3G demonstrated the concentration-dependent inhibition of the SARS-CoV-2 PLpro in an in vitro enzymatic inhibition assay within a micromolar range [72,73].

The effects of C3G and hydrochlorothiazide (HCT) on T-cell function were investigated in spontaneously hypertensive rats (SHRs). Male SHRs and Wistar-Kyoto (WKY) rats were given water, C3G (10 mg/kg per day), HCT (10 mg/kg per day), or a combination of both for 15 weeks. SHRs showed lower proportions of specific helper T-cells (CD62Llo, CD62L-, and CD25+) than WKY rats. C3G increased specific T-cell proportions (CD62Llo and CD62L-) in SHRs while HCT had mixed effects (higher CD62Lhi and CD62Llo and lower CD62L-). C3G increased TNF-α and IFN-γ concentrations while HCT decreased them. This suggests that C3G positively affects T-cell function while HCT further suppresses it in SHRs [74]. In rheumatoid arthritis (RA), fibroblast-like synoviocytes (FLS) contribute to synovitis, chronic inflammation, and joint damage. Cyanidin effectively inhibited the IL-17A-induced migration and proliferation of FLS cells in rats with adjuvant-induced arthritis (AA). Cyanidin also reduced the overexpression of IL-17 receptor A (IL-17RA) and downregulated IL-17A-dependent factors. Additionally, cyanidin modulated JAK/STAT-3 signaling and activated the PIAS3 protein, suppressing STAT-3-specific transcriptional activation. In AA rats, cyanidin treatment alleviated clinical symptoms, synovial pannus growth, immune cell infiltration, and bone erosion. It also reduced the serum levels of IL-23 and GM-CSF and decreased the level of *p*-STAT-3 protein in the synovial tissue [52]. In a study on bovine type II collagen-induced arthritis (CIA) rats, C3G was administered via tail vein injections. C3G reduced CD38+ cell proportion, suppressed rheumatoid arthritis synovial fibroblast (RASF) proliferation, and decreased proinflammatory cytokine secretion. C3G also increased regulatory T (Treg) cell proportion and upregulated Sirtuin 6 (SIRT6) expression, suppressing natural killer group 2D (NKG2D) expression in CD38+ NK cells. This elevated TNF-α secretion and decreased IFN-γ secretion in CD38+ NK cells, promoting mononuclear cell differentiation into Treg cells. The inhibition of Treg cell differentiation by CD38+ NK cells may contribute to the immune imbalance observed in RA and CIA [75].

Delphinidin 3-*O*-glucoside (D3G) and delphinidin (DC) were found to inhibit human colorectal cancer cells (HCT-116 and HT-29) at concentrations ranging from 100 to 600 µg/mL. The IC_50_ values for D3G were 329 µg/mL (HCT-116) and >600 µg/mL (HT-29); meanwhile, for DC, they were 242 µg/mL (HCT-116) and >600 µg/mL (HT-29). C3G, D3G, DC, and D3G-rich extracts also reduced PD-L1 protein expression in HCT-116 cells. C3G specifically decreased PD-L1 fluorescence intensity by 39%. Moreover, C3G reduced PD-1 expression in peripheral blood mononuclear cells by 41% in monoculture and by 39% and 26% in co-culture with HCT-116 and HT-29 cells, respectively. D3G also reduced PD-1 expression by 50% and 51% in co-culture with HCT-116 and HT-29 cells, respectively [76]. CAN supplementation, specifically glycosides of delphinidin and cyanidin, alleviated the negative effects of a high-fat diet (HFD) in rats. The HFD caused intestinal permeabilization, endotoxemia, reduced tight junction protein expression, increased nicotinamide adenine dinucleotide phosphate (NADPH) oxidase and nitric oxide synthase (NOS)-2 expression, oxidative stress, and the activation of redox-sensitive signals. ACN supplementation increased glucagon-like peptide (GLP)-2 levels, preventing permeabilization and oxidative stress and activating signaling pathways in cell cultures. In HFD-induced obese mice, ACN restored gut microbiotal composition and MUC2 levels, protective mucin in the intestinal barrier, and the immune response [77]. An ACN-rich *Lonicera caerulea* L. fruit extract containing C3G (387.60 mg/g), cyanidin 3-rutinoside (23.62 mg/g), peonidin 3-glucoside (22.20 mg/g), and cyanidin 3,5-diglucoside (8.16 mg/g) showed significant inhibition of SMMC-7721 cell growth at concentrations of 0.1–0.8 mg/mL. The extract arrested the cell cycle at the G2/M phase, causing DNA damage and apoptosis. In H22 tumor-bearing mice, the extract, at doses of 50, 100, and 200 mg/kg bw/day for 15 days, enhanced antioxidant activity, reduced lipid peroxidation levels, and regulated immune cytokines, including IL-2, IFN-γ, and TNF-α. These findings demonstrate the potent anti-tumor effect of the ACN-rich *L. caerulea* fruit extract by maintaining redox balance and enhancing immunoregulatory activity [78].

The protective effects of C3G against TNF-α-stimulated intestinal cell-induced endothelial cell activation were investigated in an in vitro co-culture system. TNF-α induced NF-κB translocation and increased TNF-α and IL-8 gene expression in Caco-2 cells, which was reduced by C3G pre-treatment. TNF-α-stimulated Caco-2 cells activated endothelial cells, increasing E-selectin and vascular cell adhesion molecule (VCAM)-1 mRNA levels, leukocyte adhesion, and NF-κB levels in HUVECs. C3G inhibited these effects by selectively inhibiting the NF-κB pathway in epithelial cells. These findings suggest that ACNs, like C3G, may hold promise for managing chronic inflammatory gut diseases [79]. The ethanol extract (25–400 µg/mL) of phenolic-rich elderberry (*Sambucus nigra* L. subsp. *canadensis*) and specific ACNs, such as cyanidin chloride and C3G (6.25–25 μM), showed potent dose-dependent inhibition of lipopolysaccharide or IFNγ-induced reactive oxygen species (ROS) production, indicating strong anti-inflammatory and antioxidant properties [80]. C3G (1.25–5 µg/mL) suppressed the production of Th2 cytokines IL-4 and IL-13 in phorbol myristic acetate/ionomycin-activated EL-4 T cells, potentially inhibiting allergic reactions. This suppression occurred at the transcriptional level by downregulating the GATA-3 transcription factor, suggesting that C3G could be a promising anti-allergic agent targeting Th2 activation [81].

The ethanol extract from black raspberries (100 and 200 µg/mL) and its metabolites, cyanidin 3-rutinoside (10–200 μM) and quercetin-3-rutinoside (10–200 μM), exhibited immunomodulatory effects by inhibiting T-cell proliferation, limiting myeloid-derived suppressor cell expansion, and attenuating IL-6-mediated STAT3 signaling. These findings suggest that black raspberries and their components could be developed as targeted immunotherapies or drugs to inhibit specific STAT-regulated signaling pathways [82]. Novel strategies are sought to prevent relapse in adult patients with Philadelphia chromosome-positive acute lymphoblastic leukemia (Ph+ ALL). ACN-rich berry extract and imatinib effectively inhibited leukemia cell proliferation at more than 6-mercaptopurine. However, the most promising results were observed with DNA vaccination and the berry extract, leading to higher survival rates and reduced relapse rates. This suggests its potential to enhance leukemia-specific immunity and be a maintenance therapy for other malignancies [83]. Two polyphenol-rich juices were investigated in a randomized crossover study involving healthy men on a low-polyphenol diet. These juices contained cyanidin glycosides (236 mg) and epigallocatechin gallate (226 mg) and showed positive effects, including improved antioxidant status, reduced oxidative DNA damage, and enhanced immune cell functions. Interestingly, there was a time delay between juice intake and the observed reductions in oxidative DNA damage and increases in interleukin-2 secretion [84].

Proanthocyanidins found in lingonberry enhance anti-inflammatory macrophage (M2) activation and inhibit proinflammatory M1 activation in mouse and human macrophages. They also promote STAT6 phosphorylation, indicating their potential for anti-inflammatory effects and suggesting that they may provide a beneficial addition to a healthy diet [85]. D3G has been found to stimulate mesenchymal stem cell (MSC) proliferation and enhance the production of anti-inflammatory cytokines, such as IL-10 and TGF-β while reducing NF-κB expression. Conditioned media from MSCs treated with D3G inhibit macrophage metabolism and reduce the production of proinflammatory cytokines (IL-1β, IL-12, and TNF-α) while increasing IL-10, demonstrating their potential to modify immunoregulatory properties and promote IL-10 production by macrophages [86]. In a study using a C57BL/6 mice model treated with azoxymethane (AOM) and dextran sodium sulfate (DSS) for 11 weeks, researchers compared the effects of black lentil (BL) water with delphinidin 3-*O*-(2-*O*-β-d-glucopyranosyl-α-l-arabinopyranoside) (D3G)-rich lentil extract (41 mg/kg body weight) on tumor development, inflammation, and the immune response. The AOM/DSS + BL group displayed a lower disease activity index with fewer neoplasms than the AOM/DSS control group. Additionally, proinflammatory cytokines, such as IL-1β and IL-6, were downregulated in the colon mucosa of the AOM/DSS + BL group. Both BL and D3G-rich extracts exhibited anti-inflammatory and pro-immune effects while BL also prevented neoplasia growth [87].

ACN supplementation (glycosides of cyanidin and delphinidin) was found to combat obesity, dyslipidemia, insulin resistance, and steatosis in mice on a high-fat diet (HFD) for 14 weeks. The HFD caused intestinal permeabilization, endotoxemia, and reduced expression of tight junction proteins (occludin, ZO-1, and claudin-1) in the ileum, as well as increasing NADPH oxidase (NOX1 and NOX4), NOS2, oxidative stress, and the activation of redox-sensitive signals (NF-κB and ERK1/2). ACN supplementation reversed these effects, raised GLP-2 levels, prevented permeabilization in vitro, and restored gut microbiotal composition and MUC2 levels (protective mucin). In vitro experiments showed that ACNs prevented Caco-2 monolayer permeabilization induced by TNF-α. ACNs also inhibited the upregulation of NOX1/4, oxidative stress, and the activation of NF-κB and ERK in inflammatory processes [76]. Delphinidin chloride and its degradation product, gallic acid, promoted the differentiation of Tregs and enhanced immune response control. Induced Tregs secreted more immunosuppressive proteins and demonstrated increased functionality. In an allograft model, treatment with these compounds reduced activated T cells by promoting Treg differentiation. Delphinidin and gallic acid are promising candidates for treating immune cell activation-related diseases, including autoimmune conditions, and could be relevant in immune-system-associated food products [88]. Delphinidin inhibited the proliferation and differentiation of specific T-lymphocytes (Th1, Th17, and Treg) without affecting Th2 subsets. The inhibition of T-cell activity was attributed to its ability to inhibit calcium signaling, histone deacetylase (HDAC), NFAT activations, and ERK1/2 activation through the estrogen receptor (ER)-α pathway [89].

Delphinidin has shown promising results in both in vitro and in vivo experiments, effectively inhibiting proliferation, the activation of pathway components, and the secretion of proinflammatory cytokines/chemokines, ultimately improving skin conditions in a mouse model of psoriasis. These findings highlight the potential of delphinidin as a modulator of the phosphoinositide 3-kinase (PI3K)/protein kinase B (AKT)/mammalian target of rapamycin (mTOR) pathway for psoriasis treatment [90]. In prostate cancer, the combination of delphinidin and tumor-necrosis-factor-related apoptosis-inducing ligand (TRAIL) has been found to activate caspase pathways, relying on the activation of death receptor 5 (DR5) and cleavage of HDAC3. This exciting finding suggests that delphinidin could be used for prostate cancer chemoprevention by enhancing TRAIL-induced apoptosis [91]. Delphinidin effectively suppresses inflammatory signaling by blocking the acetylation of p65, leading to the accumulation of p65 in the cytosol and the nuclear localization of IKBα. This inhibitory effect prevents the expression of genes regulated by NF-κB and the production of proinflammatory cytokines, making delphinidin a potential therapeutic candidate for preventing inflammatory arthritis [92].

Resveratrol and peonidin 3-*O*-glucoside (Peo3G)-rich red grape vine leaf extract exhibit preventive properties against DNA entry into macrophages, leading to the inhibition of apoptosis-associated speck-like protein containing a caspase activation and recruitment domain (CARD) (ASC) oligomerization, caspase-1 activation, and the secretion of IL-1β and IL-18. In a mouse model of psoriasis induced by imiquimod, the extract reduces psoriasis-related inflammation, including caspase-1 activation, IL-1β maturation, IL-17 production, and overall disease severity [93]. Another ACN, malvidin 3-*O*-glucoside (M3G), has been investigated for its therapeutic potential in reducing inflammasome-induced inflammation. The research shows that M3G targets various types of inflammasomes, such as nucleotide-binding domain, leucine-rich–containing family, pyrin domain–containing-3 (NLRP3), NLR family CARD domain containing 4 (NLRC4), and those absent in melanoma 2 (AIM2), resulting in decreased levels of caspase-1 and IL-1β proteins in microglia and the brain. Additionally, M3G exhibits beneficial effects in alleviating anxiety and depression symptoms and mitigating bacterial-mediated inflammation and stress-induced inflammasome-mediated innate responses [94]. Furthermore, M3G promotes resilience by modulating brain synaptic plasticity and peripheral inflammation. In a mouse model of increased systemic inflammation caused by transplanting stress-susceptible hematopoietic progenitor cells, M3G, in combination with deep hypothermic circulatory arrest (DHCA), reduces depression-like symptoms. This reduction is achieved by enhancing the histone acetylation of the regulatory sequences of the Rac1 gene, thereby modulating synaptic plasticity [95].

The immunomodulatory activities of ACNs (reported in vitro, in vivo, and in human studies) have been summarized in Table 1.

## 4. Gut Microbiota in the Immunity and Metabolism of ACNs

Gut health has become a global concern due to its significant impact on human well-being; it is often called “the second brain” [96]. The human body houses a substantial number of bacteria, estimated to be 3.9 × 10^13^ in the entire body of a 70 kg “reference man” [97]. The gut microbiota includes *Bacteroides*, *Clostridium*, *Prevotella*, *Eubacterium*, *Ruminococcus*, *Fusobacterium*, *Peptococcus*, and *Bifidobacterium,* with a smaller presence of *Escherichia* and *Lactobacillus* [98,99]. This microbiota plays a vital role in the innate and adaptive immune system, which ensures a symbiotic relationship with the microbiota [100]. The gut microbiota plays a significant role in metabolizing ACNs into phenolic acids and aldehyde metabolites, primarily through specific bacteria in the large intestine. These metabolites can influence various physiological processes [101,102,103]. Conversely, ACNs contribute to the enhancement of gut health by modulating the function of the gut barrier and promoting the colonization of beneficial bacterial species. This dynamic interplay leads to heightened protection against pathogens, optimized nutrient metabolism, and an overall reinforcement of the immune response [57].

A significant portion of dietary ACNs remains unabsorbed, arriving at the colon without absorption during digestion, then, interacting with gut microbiota and undergoing degradation, potentially increasing their bioavailability through bacterial or chemical processes. Notably, certain bacteria in the colon, such as *Bacteroides* spp., *Enterococcus casseliflavus*, *Eubacterium* spp., and *Clostridium* spp., play a key role in this process by producing enzymes, such as *β*-glucosidase, *α*-galactosidase, and *α*-mannosidase, that break down ACNs [104,105]. In the colon, unabsorbed ACNs are hydrolyzed by microbial enzymes capable of cleaving sugar linkages and releasing ACN aglycones [106,107], which are then further metabolized into phloroglucinol aldehyde (e.g., 2,4,6-trohydroxybenzaldehyde, 3,4-dihydroxy benzaldehyde) and phenolic acids (PAs) derived from phloroglucinol (A ring) and benzoic acids (B ring) [106]. Research findings have identified distinct metabolic pathways associated with the fermentation of various anthocyanidins (aglycones), leading to diverse phenolic compounds. For example, the fermentation of cyanidin-3-*O*-rutinoside and C3G yields PCA, *p*-coumaric acid, and phloroglucinol aldehyde [108]. Similarly, delphinidin-3-*O*-rutinoside undergoes elaboration, resulting in gallic acid, syringic acid, and phloroglucinol aldehyde [108]. M3G is converted to syringic acid [109] while pelargonidin-3-*O*-glucoside (Pel3G) is metabolized into 4-hydroxybenzoic acid [110]. The initial conversion of petunidin yields 3-*O*-methyl gallic acid; subsequent *O*-demethylation leads to the production of gallic acid [110]. Furthermore, peonidin undergoes sequential transformations, first into vanillic acid (3-methoxy-4-hydroxybenzoic acid) and then into PCA [109]. In red radish, acylated ACNs, such as pelargonidin-3-sophorosid-5-glucoside acylated with ferulic acid and malonic acid, undergo degradation, resulting in the formation of 4-hydroxybenzoic acid, *p*-coumaric acid, ferulic acid, and caffeic acid [111].

A high-fat, high-sucrose diet induced metabolic and inflammatory changes in mice, resulting in decreased levels of beneficial gut bacteria (*Bacteroidetes* and *Muribaculaceae*) and propionate. However, supplementation with Saskatoon berry powder, C3G, or PCA mitigated these effects and increased fecal *Muribaculaceae* and propionate [112]. Another study using an Apc^Min/+^ mouse model compared the effectiveness of the dietary administration of PCA and black raspberries (BRB) in preventing colorectal cancer. Both 5% BRBs and 500 ppm PCA-supplemented diets significantly reduced the development of adenoma in the small intestine and colon. They promoted a shift to anti-inflammatory bacterial profiles, suggesting potential benefits for colorectal cancer patients. Interestingly, 500 ppm PCA increased IFN-γ and SMAD4 (mothers against decapentaplegic homolog 4) in primary cultured human natural killer cells. In comparison, 1000 ppm PCA did not show the same effects [113]. Furthermore, a study investigated the long-lasting cardioprotective effect of dietary ACNs by examining gene expression, histology, and resistance to ischemia/reperfusion in mice hearts after a month following the cessation of a C3G-enriched diet. The results indicate that the cardioprotective benefits persisted, independent of immune responses, and were attributed to changes in the gut microbiota induced by the C3G-enriched diet [69].

C3G also alleviated polystyrene (PS)-induced toxicities in Caco-2 cells and *Caenorhabditis elegans* by promoting autophagy and discharge. In C57BL/6 mice, C3G supplementation reduced tissue accumulation, promoted fecal PS discharge, alleviated oxidative stress and inflammation caused by PS, and modulated PS-associated gut microbiome perturbations, indicating its potential protective role against PS-induced toxicity and gut dysbiosis [114]. In a study investigating the potential of purple sweet potato ACN extract in modulating the gut microbiota in a dextran sulfate sodium (DSS)-induced chronic colitis mouse model, treatment with the extract prevented the loss of beneficial bacteria (*Bifidobacterium* and *Lactobacillus*), inhibited increases in harmful bacteria (*Gammaproteobacteria* and *Helicobacter*), and maintained colonic tight junction protein expression and architecture, resulting in attenuated intestinal inflammation. These findings suggest that the extract could be an effective and safe treatment for chronic colitis [115]. Combining bilberry ACN with chitosan and low molecular citrus pectin was proposed to enhance digestive stability and modulate the microbiome for programmed cell death-ligand 1 (PD-L1) blockade treatment. This combo enriches subdominant species, increases butyrate concentration, enhances intra-tumoral CD8+ T cell infiltration, and restores gut microbiome diversity, improving control over tumor growth. These findings indicate potential opportunities for probiotic combos to enhance the therapeutic efficiency of immune checkpoint inhibitors through gut microbiome manipulation [116].

Bilberry ACN supplementation attenuated western diet-induced serum aspartate aminotransferase, alanine transaminase, low-density lipoprotein cholesterol levels, liver fat content, thiobarbituric acid reactive substances, and alpha-smooth muscle actin. Additionally, it modified the gut microbiota by reducing the Firmicutes/Bacteroidetes ratio and increasing the relative abundance of *Akkermansia* spp. and *Parabacteroides* spp., which suggests it may have the potential to ameliorate nonalcoholic fatty liver disease by addressing dyslipidemia and gut microbiome dysbiosis [117]. M3G ingestion improved histopathological scores, enhanced IL-10 expression, promoted beneficial microbial interactions, and reduced the abundance of pathogenic bacteria and inflammatory mediators, demonstrating its unique mechanism of action on the gut microbiome compared to a whole blueberry in the DSS-induced colitis mouse model [118]. C3G has shown the potential to support gut integrity and overall health by producing phenolic compounds that combat gut inflammation and oxidative stress. These metabolites activate nuclear factor erythroid 2-related factor 2 (Nrf2) and influence TGFβ-activated kinase 1 (TAK1)-mediated mitogen-activated protein kinase (MAKP) and sphingosine kinase (SphK)/sphingosine-1-phosphate(S1P)-mediated NF-κB pathways, collectively reducing inflammation and creating an optimal environment for gut metabolism [119].

In a study investigating the effects of six berries, each containing different ACN profiles, on metabolic risk factors in a mouse model of polygenic obesity, the biological activities varied based on their delphinidin/malvidin-versus cyanidin-type ACNs, which were influenced by their structure and metabolism in the gut. Berry consumption shifted gastrointestinal bacterial communities towards obligate anaerobes, reducing luminal oxygen and oxidative stress [120]. A study investigated the distribution of black currant ACNs and their metabolites in lean and diet-induced obese mice with intact or disrupted gut microbiomes. Daily consumption of a black currant extract-supplemented diet improved glucose metabolism and reduced weight gain in mice with an intact gut microbiome, but this was not the case in those with a disrupted one. The results highlight the role of the gut microbiome and ACN aglycone type in modulating the protective effects of ACNs against obesity and insulin resistance [121]. Another study examined the metabolism of M3G, gallic acid, and a mixture of ACNs by gut bacteria. Most ACNs disappeared after 5 h of incubation while gallic acid degraded almost completely after 24 h. M3G resulted in the formation of syringic acid; whereas, the mixture of ACNs led to the formation of gallic, syringic, and *p*-coumaric acids. Furthermore, the tested ACNs promoted the growth of *Bifidobacterium* spp. and *Lactobacillus*–*Enterococcus* spp. [122]. The findings from all the studies above collectively support the potential of ACNs as promising natural interventions to enhance gut health and contribute to preventing and alleviating diseases.

## 5. Nutraceutical Potential of ACNs

**Figure 3:**
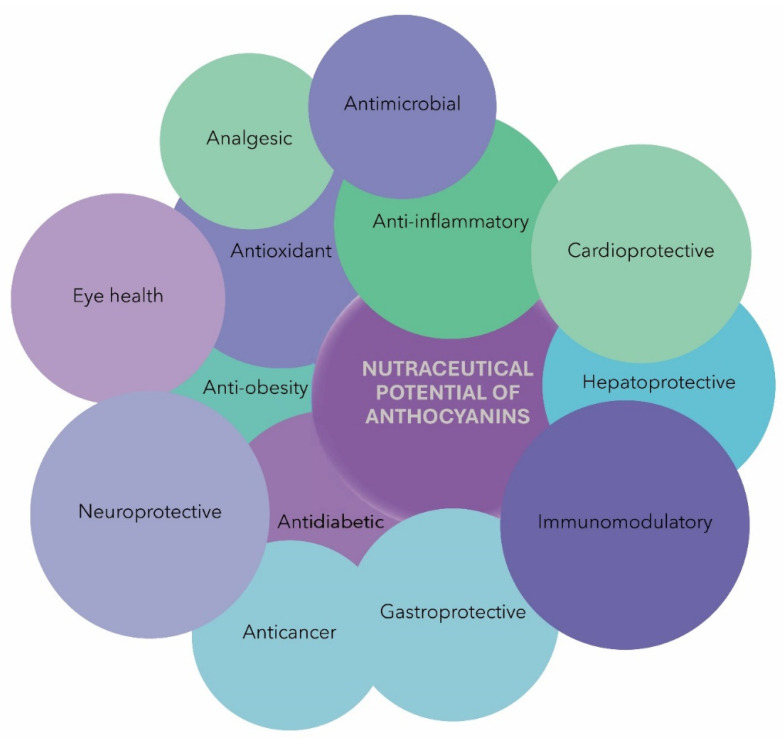
Nutraceutical potential of anthocyanins based on reported studies.

ACNs have gained considerable attention in the nutraceutical industry, primarily for their role as natural colorants and antioxidants (Figure 3). ACN chalcones and quinoidal bases have been reported to be efficient antioxidants, with the glycosylated B-ring structure of ACNs enhancing their antioxidant activity, especially in terms of ortho-hydroxylation and methoxylation [33,48,123]. However, anthocyanidin, the aglycone form of ACNs, exhibits higher ORAC values due to its greater stability and reactivity [124]. The acylation of ACNs with phenolic acids significantly increases their antioxidant activity; diacylation enhances their activity further while 5-glycosylation reduces said activity [124,125]. Studies have shown that various ACNs extracted from plants possess antioxidative properties. C3G, D3G, and Peo3G from black bean seed coats exhibit strong antioxidative activity and reduce malondialdehyde formation by ultraviolet B (UV-B) irradiation [126]. Delphinidin and D3G show the highest inhibitory effect on lipid peroxidation and O^2•−^ scavenging activity. On the other hand, cyanidin and C3G demonstrate the highest inhibitory effect on copper (II)-induced low-density lipoprotein (LDL) oxidation, while delphinidin has intermediate efficacy [127]. Moreover, ACNs activate Nrf2 and antioxidant enzymes, such as superoxide dismutase (SOD), catalase (CAT), and glutathione peroxidase (GPx), enhancing their enzymatic activity [128,129]. In addition to their antioxidant effects, ACNs demonstrate potent activity against inflammation and associated disease conditions [50]. The imbalance between proinflammatory molecules and anti-inflammatory mediators leads to chronic inflammation, which is linked to various chronic diseases, such as neurodegenerative disease, arthritis, allergies, liver disease, diabetes, obesity, cardiovascular disease, and cancer [130]. Several studies showed that the ACNs suppress the activation of NF-κB [131] and MAPK signaling cascades [64], inhibiting the production of proinflammatory cytokines and enzymes, such as COX-2 [132]. ACNs directly inhibit COX-1 and COX-2 enzymes, thus reducing the production of prostaglandin E_2_ (PGE_2_) [132]. Moreover, ACNs also suppress NLRP3 inflammasomes, a complex involved in inflammatory responses [133,134].

Furthermore, ACNs exhibit promising potential as oral hepatoprotective agents against chemically-induced liver damage. They effectively combat liver lipid accumulation, inhibit lipogenesis, promote lipolysis, and counteract oxidative stress. They decrease liver enzyme levels, enhance antioxidant enzymes, and suppress the expression of COX-2 and iNOS, suggesting their potential therapeutic use in treating oxidative stress in the liver and its associated diseases [135]. C3G–lauric acid ester displayed enhanced antioxidant activity compared to its precursor C3G; safeguarding cells through the regulation of oxidative stress and PI3K/Akt-mediated Nrf2–heme oxygenase 1 (HO-1)/nicotinamide adenine dinucleotide (phosphate) reduced quinone oxidoreductase (NQO1) pathway activation, highlighting the potential of acylation for improved antioxidative effects and stability [136]. *Myrciaria jaboticaba* (Vell.) O.Berg and *Syzygium cumini* (L.) Skeels peel powders exhibited protective effects against liver fibrosis and hepatocarcinogenesis while *Syzygium malaccense* (L.) Merr. and L.M.Perry showed antioxidative benefits, emphasizing the anthocyanin profile effect [137]. *Lonicera caerulea* L. anthocyanin extract demonstrated hepatoprotective potential against alcoholic steatohepatitis through AMP-activated protein kinase (AMPK)-mediated anti-inflammatory and lipid-reducing pathways [138]. Bilberry fruit anthocyanin extract showed hepatoprotective effects against acute liver failure, reducing damage markers, oxidative stress, inflammation, and necrosis; these effects are attributed to antioxidative and anti-inflammatory properties [139].

Due to a high lipid content and energy demand, the central nervous system (CNS), particularly the brain, is highly vulnerable to excessive ROS [140]. ROS can be generated internally during cellular respiration and externally through environmental factors, such as pollution, smoking, UV-B radiation, and infections [141]. An excessive inflammatory response in the CNS can contribute to neuronal apoptosis and the progression of neurodegenerative diseases [142]. In this context, ACNs found in various fruits and vegetables have shown neuroprotective effects in preclinical models of neurodegenerative diseases and cerebral ischemia [143]. ACNs exert their neuroprotective effects through multiple mechanisms. They act as direct scavengers of ROS [144], enhance antioxidant response pathways [145], and inhibit neuroinflammation by modulating specific signaling pathways [146,147,148]. Studies in animal models have demonstrated that ACNs can prevent cognitive decline, delay the onset of neurodegenerative diseases, such as Alzheimer’s [149] and Parkinson’s [150], and reduce brain damage in cerebral ischemia. In a randomized controlled trial involving twelve participants with mild cognitive impairment, older adults demonstrated enhanced learning and memory capacity after 12 weeks of grape juice supplementation, compared to the placebo group [151]. Similarly, in another study, a 12-week supplementation of blueberry juice in the same group improved memory function [152,153]. These findings suggest that ACNs could be a potential co-adjuvant therapy to pharmacological treatments or a preventive strategy to reduce drug dosage and adverse effects in managing neurodegenerative diseases, cerebral ischemia, and cognition.

Dietary ACNs have demonstrated beneficial effects in protecting against cardiovascular diseases (CVDs) in animal and human studies [153]. Animal studies have shown that ACNs can prevent hypertriglyceridemia, hypercholesterolemia, and platelet hyperactivity induced by high-fat or high-fructose diets, while also protecting cardiac tissue against oxidative stress in ischemia/reperfusion conditions [154]. Human intervention studies have revealed that berry ACN intake can increase high-density lipoprotein (HDL)-cholesterol and reduce LDL-cholesterol, triglycerides, blood pressure, and inflammatory markers [155,156]. Furthermore, higher ACN intake has been associated with a reduced risk of myocardial infarction and decreased incidence of coronary heart disease and CVD-related mortality [157,158]. The cardioprotective mechanisms of ACNs include increasing plasma antioxidant capacity and nitric oxide levels, reducing LDL oxidation and platelet aggregation, and enhancing glutathione and omega-3 fatty acid synthesis. Cardiovascular diseases, including myocardial infarction, ischemic heart disease, and stroke, are major causes of mortality globally. Atherosclerosis, driven by oxidized low-density lipoproteins (ox-LDL), plays a key role in these diseases. ACNs have been found to protect against atherosclerosis in animal models by reducing the formation of atherosclerotic plaques and improving dyslipidemia. ACNs also prevent LDL-cholesterol oxidation [159], reduce inflammation [160], and decrease the expression of adhesion molecules in the aorta [160,161], leading to decreased leukocyte infiltration and proinflammatory cytokines. Additionally, ACNs have shown cardioprotective effects in animal models of myocardial infarction and chemotherapeutic drug-induced cardiotoxicity [162].

Obesity is a significant global health issue associated with numerous metabolic disorders. Studies have shown that dietary ACNs can have beneficial effects in combating obesity. One mechanism through which ACNs act as anti-obesity agents is by increasing energy expenditure [163]. Berries rich in ACNs, such as petunidin and malvidin, were found to effectively reduce the metabolic damage caused by a high-fat diet by boosting energy expenditure and reducing mitochondrial dysfunction in adipose tissue [164]. Moreover, ACNs have been found to modulate AMPK, a key regulator of energy balance [165,166]. By activating AMPK, ACNs can promote mitochondrial biogenesis, reduce lipid metabolism, increase fatty acid oxidation, and improve glucose metabolism. This activation of AMPK has been associated with improved glucose uptake in skeletal muscle, decreased glucose production in the liver, and decreased liver lipid content and serum lipoproteins. Furthermore, dietary ACNs have been shown to affect lipid metabolism by downregulating key molecules, such as fatty acid synthase and sterol-regulatory element-binding proteins. ACNs from different sources were found to reduce hyperglycemia and inhibit hepatic lipogenesis, potentially suppressing inflammation in obese individuals [167,168].

In addition to their effects on energy expenditure and lipid metabolism, ACNs have been linked to the modulation of the gut microbiota [169]. Changes in the gut microbiota in obese individuals can contribute to metabolic disorders and ACNs have been studied for their potential role in regulating the gut microbiota to combat obesity-related conditions. Moreover, ACNs have demonstrated promising results in protecting pancreatic β cells, improving insulin resistance, and reducing cholesterol levels in diabetic subjects [170]. ACN-rich beverages have also positively affected the postprandial glycemic response and insulin excretion [171]. Numerous in vitro and in vivo studies have consistently supported the potential anti-obesity effects of ACNs, demonstrating their ability to inhibit fat accumulation [172], improve lipid profiles [168], and reduce adipocyte hypertrophy [173]. Furthermore, ACNs have shown anti-inflammatory properties by suppressing NF-κB signaling and promoting an anti-inflammatory phenotype in adipose tissue macrophages [174]. Thus, ACNs present a promising natural approach to combat obesity and related metabolic disorders. The mechanisms through which ACNs exert their anti-obesity effects involve increased energy expenditure, modulation of AMPK, lipid metabolism, gut microbiota, and anti-inflammatory properties.

ACNs have emerged as potential antitumoral agents, attracting significant interest in recent decades [175,176]. Cancer is characterized by the uncontrolled cell proliferation, resistance to apoptosis and cell migration, often involving the dysregulation of pathways, such as Notch, Wnt/β-catenin, NF-κB, and MAPK [177]. Dietary ACNs extracted from berries have been found to modulate the levels of proteins involved in these pathways, leading to cell growth inhibition. Notably, ACN mixtures have shown enhanced effects compared to single purified ACNs, indicating overlapping pathways in cell growth inhibition [178]. ACNs exhibit diverse mechanisms for the prevention and inhibition of cancer, targeting pathways related to cell survival, proliferation, apoptosis, inflammation, and angiogenesis. They have been shown to suppress NF-κB [179], PI3K/Akt [180,181], and Akt/mTOR [182] signaling pathways. ACNs can also inhibit tumor growth, promote apoptosis and autophagy in cancer cells, and inhibit angiogenesis and metastatic migration [183]. In vitro and in vivo studies have demonstrated the anticancer potential of ACNs from various sources, including berry extracts [78,184] and pure ACNs, such as delphinidin [185] and cyanidin 3-rutinoside [181]. These findings suggest that ACNs hold promise regarding potential anticancer properties, making them a subject of considerable research and exploration in cancer therapy and prevention.

ACN pigments found in various berries are beneficial for maintaining good vision and have been associated with improved night vision [48,186]. Berries rich in ACNs have long been known for their positive effects on eye health. For instance, administering bilberry extract, containing about 39% ACNs, to mice has been shown to protect photoreceptor cell function during retinal inflammation [187]. In a study with 132 patients suffering from normal-tension glaucoma, daily supplementation with two capsules containing 60.0 mg ACNs from bilberry resulted in improved visual function [188]. Additionally, other berries, like blackcurrant [189] and purple corn seed [190], have shown a protective effect on eyesight, with blackcurrant ACN supplementation increasing ocular blood flow in patients with open-angle glaucoma and purple corn seed extract reducing lens opacity and malonaldehyde levels. Furthermore, ACNs from black soybean seed coats have effectively prevented retinal degeneration and suppressed human lens epithelial cell death under oxidative stress [191].

ACNs exhibit potent antimicrobial properties against a wide range of microorganisms, particularly in inhibiting the growth of food-borne pathogens. Their antimicrobial effects are attributed to various mechanisms, including cell wall, membrane, and intercellular matrix damage [175]. For instance, maqui berry extracts have shown antibacterial activity, being highly effective in working against *Aeromonas hydrophilia* and *Listeria innocua* [192], commonly associated with refrigerated foods and used as indicators of pathogenic or spoilage microorganisms [193]. Cranberry extract also demonstrates antibacterial activity against certain resistant strains of *Enterococcus faecium*, *Pseudomonas aeruginosa*, *Staphylococcus aureus*, and *Escherichia coli*, with its effect not solely due to low pH but rather to specific bioactive components, such as ACNs and flavonols [194]. ACN-rich extracts from various fruits, like blueberry, raspberry, blackcurrant, and strawberry, display inhibitory effects on gram-negative bacteria but not on gram-positive bacteria [195], likely due to differences in cell wall structures. These antimicrobial actions are likely a result of synergistic effects from various phytochemicals in the extracts, including ACNs, weak organic acids, phenolic acids, and other chemical forms [196]. Hence, the complex nature of these compounds necessitates further comprehensive analysis of their antimicrobial potential.

From a nutraceutical point of view, ACNs are highly valued in the food and supplement industry for their diverse health benefits. As natural colorants, they add vibrant hues to foods and beverages. Other than their immunomodulatory potential, ACNs possess potent antioxidant properties, neutralizing free radicals and protecting cells from oxidative damage. Moreover, they exhibit anti-inflammatory action by inhibiting pathways involved in inflammation and activating antioxidant enzymes. ACNs have shown neuroprotective effects, making them potential candidates for supporting brain health. They also protect against cardiovascular diseases by improving lipid profiles and reducing blood pressure. In obesity, ACNs are linked to increased energy expenditure and improved lipid metabolism. They demonstrate potential as antitumoral agents by targeting pathways involved in cancer cell growth. Additionally, ACNs contribute to maintaining good vision and display antimicrobial properties against food-borne pathogens. Thus, these compounds hold great promise as natural compounds with multifaceted health benefits, making them valuable in nutraceutical products for promoting overall well-being and preventing chronic diseases. As nutraceuticals, the bioavailability of ACNs is the key factor for viable biological action. The low bioavailability of ACNs causes low absorption of these compounds into the circulatory system and a high excretion rate of ACNs in urine and feces, thus reducing the efficacy of ACN bioactivity.

A considerable body of research has been devoted to the assessment of the biological effects of ACNs, including their immune modulation potential. A range of signaling pathways and cellular processes are involved in the immune modulatory activities of ACNs; these may provide potential therapeutic targets and strategies for the improvement of immune-mediated disorders in the future. However, much remains to be elucidated before their application in clinical settings. The metabolites of ACNs need an in-depth assessment of their potential to modulate immune processes as ACNs are transported in blood and urine, primarily as metabolites. In addition, the nutraceutical potential of ACNs has mostly been demonstrated using in vitro and in vivo experimental models; however, their low bioavailability is a key obstacle in achieving the desired biological effects. The development of novel approaches is required, so as to enhance the bioavailability of ACNs in human subjects.

In addition to food bioactives, the extensive literature has reported the potential biological effects of fresh fruits and vegetables, thanks to the presence of polyphenols [197,198]. As recommended by the World Health Organization (WHO), five to eight portions (i.e., 400–600 g) of fruits and vegetables may aid in the reduction of the risk for cardiometabolic disorders, poor cognitive performance, and cancer, among others. Some of these studies recommend the consumption of whole fruits and vegetables rather than isolated constituents because of the synergistic or additive effects of bioactive compounds [198,199]. However, in some cases, the desired biological effects of fresh fruits and vegetables may be masked by the presence of some co-components in the food matrix, for instance, saccharides or proteins [200,201]; thus, it is a need to precipitate out the interfering compounds present in fruits and vegetables, as suggested by Ullah et al. while assessing the potential effects of plum fruit pulp on metabolic syndrome risk factors [200].

## 6. Factors Influencing Pharmacokinetics of ACNs

The pharmacokinetics of ACNs are influenced by various factors, including the site of administration, chemical structure, pH, temperature, food matrix, and gut microbiota [107,202]. Interactions between ACNs and salivary proteins within the oral cavity can potentially lead to the degradation of these compounds [203]. Both dietary intake and binding with salivary proteins can reduce can levels in the mouth [204,205]. ACN stability is also impacted by the composition of the food matrix and the molecular structure, with higher B-ring hydroxylation leading to decreased stability [206,207,208]. ACNs exhibit sensitivity to oral pH levels within the range of 6.0 to 7.0, as well as variations in temperature. Some conversions occur under neutral to basic pH conditions [207,209]. For instance, approximately 30% of C3G can convert into its chalcone form under these conditions [207].

Upon oral consumption, ACNs enter the stomach, where the acidic pH (ranging from 1.5 to 5.0) impacts their stability [210]. In this environment, ACNs can either be absorbed or progress to the small intestine for further processing before entering the bloodstream. Studies on rats have demonstrated the presence of ACNs in both systemic and portal plasma shortly after ingestion, suggesting stomach absorption. A 14-week experiment involving 32 rats, fed diets containing chokeberry, bilberry, and grape extracts, revealed plasma ACN levels below 2 μmol/L, with higher levels of metabolites being presumed. Urine analysis showed varying concentrations of intact ACNs and methylated derivatives (e.g., 17.4 nmol/L for bilberry and 52.6 nmol/L for chokeberry) [211]. Approximately 75–97% of total ACNs were recovered from the gastrointestinal tracts of rats within 30–120 min following oral intake [212]. In vitro digestion studies indicate a 75–88% ACN recovery rate under gastric conditions, underscoring the limited degradation of ACNs by gastric enzymes [101,213]. Evidence suggests that ACNs likely traverse cell membranes through active transport mechanisms owing to their intricate structure [25,214]. Membrane carriers, such as organic anion carriers, like bilitranslocase and glucose transporters (GLUT), participate in this process [110,215,216]. ACN absorption involves GLUT1 and GLUT3, along with other transporters [214]. At gastric pH levels ranging from 1.5 to 4, ACNs maintain their glycoside form [217,218].

ACNs experience significant degradation within the intestinal tract due to elevated pH levels (ranging from pH 5.6 to 7.9), the influence of gut microbiota, and intestinal enzyme activity. The pH within the small intestine spans from 5.0 to 7.0 while the large intestine maintains a pH of 7.0 to 8.0 [219]. The enzymatic conversion of ACNs into anthocyanidins, facilitated by β-glucosidase and lactase-phlorizin hydrolase, occurs in this environment [216]. The gastrointestinal epithelial tissues absorb several bioactive ACN derivatives, including protocatechuic acid, vanillic acid, gallic acid, and phloroglucinol aldehyde [220]. The absorption of ACNs from the small intestine occurs through active transport mechanisms involving transporters, such as the sodium-dependent glucose cotransporter (SGLT1), GLUT2, and bilitranslocase [221,222,223]. The hydrolysis of ACNs into anthocyanidins enables their passive diffusion, a process facilitated by enzymes such as β-glucosidase [216,221,223,224].

In a laboratory experiment, treatment of a Caco-2 monolayer with a 200 mg/L ACN extract for four days led to an increase in the expression of glucose transporter 2 (GLUT2) and the rate of ACN transport [225]. However, the prolonged exposure (16 h) of Caco-2 cells to berry extracts (0.125%, *w/v*) resulted in the reduced expressions of sodium-dependent glucose transporter 1 (SGLT1) and GLUT2 mRNA [226]. ACNs are predominantly absorbed via the SGLT1 and GLUT2 pathways, with their phase II metabolites (glucuronide and sulfate) also being actively absorbed through ATP-binding cassette (ABC) proteins [227]. Unabsorbed ACNs within the colon undergo enzymatic breakdown through the activity of colonic bacteria, which includes enzymes like α-galactosidase, *β*-D-glucuronidase, *β*-D-glucosidase, and *α*-rhamnosidase. These enzymatic processes result in the cleavage of glycosidic bonds within a period of between 20 min and 2 h. This cleavage yields smaller phenolic compounds, including benzaldehydes and hydroxytyrosol, as well as phenol aldehydes and phenolic acids, such as hydroxybenzoic, homovanillic, phenyl propionic, protocatechuic, syringic, gallic, and vanillic acids [33,228].

In a 48-hour human study, 44% of the ^13^C label from C3G intake was excreted in urine (5.4%), breath (6.9%), and feces (32.1%), indicating complete catabolism. Over 50% of the label remained in the body, attributed to slow clearance due to colonic metabolism and other factors [229]. After 3 h in the mice, almost 90% of the ^14^C label from oral ^14^C C3G intake had migrated to adipose and gastrointestinal tissues, decreasing to 50% in feces and 3.3% in urine after 24 h [230]. Human studies indicated a slower anthocyanin clearance than in mice, with variations in recovery percentages and plasma concentrations [229]. Methylation and glucuronidation were major in vivo C3G conjugation routes, as shown in various intervention models [229,231,232]. Liver metabolism was evident, with the biliary presence of C3G and Peo3G after oral administration to rats [233]. An intravenous (IV) administration revealed rapid fluctuations of methylated C3G in plasma, indicating absorption or decomposition [234]. Anthocyanidin stability and fate depended on conjugation possibilities [229,231,232].

In swine studies, M3G reached peak concentrations in brain tissue (4.43 pmol/g) after bilberry extract intake and malvidin glycosides dominated in various tissues of blueberry-fed pigs [235,236,237]. Mice also showed malvidin predominance in plasma and tissues after bilberry anthocyanin consumption [238]. In humans, malvidin glycosides ranked among the top urinary anthocyanin metabolites during blueberry juice consumption, alongside other C6-C3-C6 forms [233]. These findings suggest malvidin’s resistance to metabolism due to its structural attributes, supported by evidence of enzymatic methylation from petunidin to M3G [239,240]. The observed pelargonidin-based metabolite recovery is attributed to functional group removal, yielding pelargonidin glycoside and pelargonidin [241,242,243]. Urinary pelargonidin glucuronide was found, due to cyanidin dehydroxylation, in a human study of various cyanidin glycosides [231]. Pel3G formation was observed upon the IV administration of C3G [234]. Notably, blueberries lack pelargonidin glycosides; yet, pelargonidin-based metabolites were abundant in urine after long-term juice intake [233], potentially making them a possible biomarker of flavonoid-based anthocyanin intake, due to pelargonidin’s high membrane solubility [244].

One investigation administered 720 mg of anthocyanins in four healthy older women, leading to the identification of anthocyanin glycosides in both the plasma and urine. Notably, the peak plasma concentration averaged at 97.4 nmol/L after 71.3 min, displaying elimination patterns adhering to first-order kinetics with a half-life of 132.6 min. Most anthocyanins were excreted within the initial 4 h, characterized by an initial excretion rate of 77 microg/h followed by 13 microg/h subsequently [245]. Further investigations explored plasma anthocyanins in overweight adults (BMI: 26 ± 2 kg/m^2^) consuming strawberry beverages with varying meal timings. Optimal concentrations and bioavailability of pelargonidin-based anthocyanins were observed when the strawberry drink was consumed two hours prior to the meal, in contrast to during or after the meal, highlighting the mealtime-dependent impact on anthocyanin pharmacokinetics [246]. A separate study investigated the pharmacokinetics of bilberry anthocyanins and their interaction with glucose transporters (SGLT1 and GLUT2) during absorption. Rats were administered a standardized bilberry extract containing 15 distinct anthocyanins, with liquid chromatography-electrospray ionization tandem mass spectrometry (LC/ESI-MS/MS) utilized to measure absorption. Fasting significantly augmented anthocyanin bioavailability sevenfold compared to fed rats and glucose co-administration exhibited no interference with uptake. Both aglycone and sugar constituents influenced the variance in the bioavailability of the anthocyanins. Computational analysis revealed a correlation between anthocyanin absorption and recognition by glucose transporters SGLT1 and GLUT2, offering valuable insights into this complex interplay [247].

The absorption and clearance kinetics of anthocyanins report maximum plasma concentrations ranging from 1.4 to 592 nmol/L, occurring within 0.5–4 h post-consumption (doses: 68–1300 mg) [248]. Urinary excretion averages 0.03–4% of the dose, with elimination half-lives of 1.5–3 h [249]. Although anthocyanin metabolism is not fully understood, recent evidence indicates that they are primarily absorbed and transported as metabolites in human serum and urine, constituting 68–80% of urinary anthocyanin content [250,251]. These findings significantly advance our understanding of the intricate processes governing ACN absorption and cellular responses to their metabolites. Due to uncertainties surrounding ACN bioavailability, numerous researchers are actively exploring innovative strategies to enhance the stability and bioavailability of these natural compounds.

## 7. Strategies for Enhanced ACN Bioavailability

The daily consumption of ACNs can vary depending on individual factors, such as age, health status, and dietary preferences. Though anthocyanin-rich foods offer potential health benefits, there is no specific recommended daily intake (RDI) for ACNs as they are not considered essential nutrients like vitamins and minerals. Despite the numerous health benefits associated with anthocyanins, their bioavailability in the plasma is limited, with less than 6% of the initial dose being absorbed after consuming a meal rich in anthocyanins. This restricted absorption is attributed to the rapid degradation of ACNs under various external conditions, such as temperature, pH, light, oxygen, water activity, and enzymatic activity. In addition, the interaction of ACNs with other components of the food matrix (such as carbohydrates, proteins, and dietary fibers) in processed foodstuffs may also affect the release of ACNs from the food matrix, thus affecting their bioaccessibility and bioavailability. These conditions lead to a high reactivity, resulting in reduced availability for the body [252]. In the United States, the reported daily consumption of ACN pigments is around 12.5 mg per person; but, the bioavailability of these compounds in the diet is relatively poor, typically averaging between 1–2% [253]. ACN stability refers to the ability of these compounds to resist degradation and maintain their chemical structure, color, and bioactivity over time and under various environmental conditions. Improving the stability and bioavailability of ACNs could maximize their health benefits and promote their inclusion in daily diets.

Using ACNs to form a polymeric matrix of polysaccharides, proteins, or liposome nanodelivery vehicles represents a promising approach for enhancing their stability and bioavailability [254]. Polysaccharides offer several advantages, including their abundant availability, cost-effectiveness, ease of chemical modification, and biodegradability, making them favorable compared to other matrices. Proteins possess a complex bioactive structure that renders them sensitive to environmental factors, such as pH, ionic strength, and temperature changes. This sensitivity may affect their stability during delivery. Liposomes exhibit reduced stability and are prone to aggregation and phospholipid oxidation, especially when stored over extended periods [255]. These polymeric matrices serve as a protective barrier for ACNs, shielding them from factors that might otherwise degrade or inactivate them, such as heat, light, oxygen, and changes in pH. Moreover, incorporating ACNs into a polymeric matrix can facilitate their controlled release, enabling a gradual and sustained release of these bioactive compounds in the body [48]. Starch [256], chitosan [55,257], cyclodextrins [258], cellulose [259], and sodium alginate [260] are frequently employed as encapsulation materials because of their excellent biocompatibility and biodegradability. Additionally, emulsion cross-linking (e.g., ionic cross-linking, covalent cross-linking, etc.), self-assembly (e.g., layer-by-layer, self-assembled micelles, etc.), and spray drying techniques are commonly utilized to prepare polysaccharide-ACN nanosystems [255].

A study found that the incorporation of β-cyclodextrin (β-CD) slowed the degradation of C3G and reduced its deterioration during simulated in vitro digestion. This finding suggests the potential of β-CD to enhance the stability and delay the degradation of blackberry ACNs under conditions similar to the gastrointestinal tract [258]. To enhance the stability of saffron ACNs under harsh gastric conditions, ACNs were encapsulated using spray drying with β-glucan and β-CD. The resulting microcapsules successfully protected the ACNs against the stomach conditions and displayed positive attributes, such as controlled release and antioxidant behavior [261]. Through spray drying, ethanolic pomace extract was microencapsulated with whey protein isolate. In vitro testing showed that while the ACN concentration decreased after intestinal digestion for microcapsules, it remained consistent for the non-encapsulated system. This indicates the potential of microencapsulation to influence the release of ACNs during digestion [262]. A complex consisting of *Bryophyllum pinnatum* (Lam.) Oken extract and β-CD was created and integrated into an oil-in-water emulsion for topical anti-inflammatory evaluation. The incorporation of β-CD enhanced the aggregate properties and antioxidant activity of the emulsion, suggesting its potential in topical applications [263]. The microencapsulation of anthocyanin-rich extracts from Leum Pua black rice bran was achieved through spray drying with various wall materials, including maltodextrin, gum arabic, and whey protein isolate. The optimal retention of ACNs was observed with maltodextrin and maltodextrin-whey protein combinations while whey protein demonstrated high phenolic content and antioxidant activity. During in vitro digestion, microcapsules formed from pure whey protein showed the highest release of phenolic content and antioxidant activity in intestinal fluids [264].

Sour cherry skin ACNs were successfully encapsulated in whey protein isolate microcapsules, demonstrating substantial polyphenol content and facilitating controlled release in the intestine. Additionally, the encapsulated ACNs promoted the growth of *Lactobacillus casei* 431^®^, a beneficial probiotic bacterium [265]. A study investigated the impact of whey protein and citrus pectin encapsulation on ACN bioavailability and intestinal accessibility in humans. The results indicate that whey protein encapsulation influenced short-term bioavailability. In contrast, citrus pectin encapsulation enhanced intestinal accessibility, affecting the formation of degradation products in human plasma during passage through the small intestine [266]. The bioavailability of ACNs from blackberry pomace microcapsules and microcapsule-supplemented yogurt was evaluated after in vitro digestion. Three microencapsulation methods (spray drying, freeze-drying, and ionic gelation) were compared, with spray drying and ionic gelation producing stable microcapsules that improved C3G bioavailability in yogurt formulations [267]. Another study aimed to enhance the stability and bioavailability of ACNs through cyclodextrin encapsulation. Encapsulation slowed down ACN degradation and led to the formation of phenolic acids. The study also highlighted the potential of encapsulation to enhance ACN release in the colon, potentially conferring health benefits [268]. A novel approach utilized chitosan/β-lactoglobulin nanocomplexes to encapsulate ACNs for enhanced sustained release, stability, and bioavailability. The optimized nanocomplexes exhibited improved stability and bioavailability under simulated gastrointestinal conditions, showing promise for sustained release applications [269]. The encapsulation of anthocyanin-rich bilberry extract (BE) aimed to improve its stability. The study revealed that encapsulated BE exhibited a slower release and higher availability of ACNs, indicating the role of encapsulation in inhibiting early degradation in the intestinal system [270].

Researchers developed a thermally induced matrix system using strongly acidic whey protein isolate gels to encapsulate anthocyanin-rich bilberry extract. The encapsulation process influenced release kinetics, stabilization, and release characteristics, with pH-dependent interactions impacting overall stability [271]. Alginate/chitosan beads were fabricated through spray drying and external gelation methods to encapsulate ACNs, enhancing their stability and bioavailability. The study found that bead degradation and electrostatic interactions influenced ACN release, with certain beads identified as optimal microcarriers [272]. For Iranian borage ACNs, maltodextrin/modified maize starch was identified as the optimal carrier for targeted drug delivery to the intestines. Modifying starch improved its solubility and swelling behavior, improving its potential as an effective drug carrier [256]. Porous microgels derived from oxidized starch hydrolysate were created to enhance ACN delivery. These microgels exhibited efficient adsorption and release, ultimately improving ACN stability and release [273]. Chitosan nanoparticles were employed to encapsulate ACNs from black carrots, leading to pH-dependent release profiles that protected against rapid degradation within the digestive environment. This technique augmented antioxidant effectiveness, stability, and overall ACN presence [274]. A composite co-precipitation technique using chitosan and pectin was utilized to fabricate nanocarriers for ACNs, offering protection against degradation and delayed release upon exposure to intestinal fluid [275]. Cellulose nanocrystals were integrated into chitosan-based microencapsulation for blueberry ACN extracts, resulting in durable microcapsules with enhanced stability and controlled release properties [276].

Lipid-based nanocarriers encompass diverse systems that utilize lipids, such as fats and oils, providing versatility in solubilizing bioactive ingredients. They can dissolve or entrap these ingredients within the oil phase for hydrophobic components or the aqueous phase for hydrophilic ones. One notable category within this group is nanoemulsions, comprising oil, water, and surfactants/biopolymers in various forms, including single O/W or W/O nanoemulsions, double nanoemulsions (O/W/O or W/O/W), Pickering nanoemulsions stabilized by biopolymer nanoparticles, and structural nanoemulsions stabilized by single or double layers of biopolymer coatings [277,278,279]. Another significant group of lipid-based nanocarriers is nanoliposomes, which consist of phospholipids, oils, and varying solvents. Nanoliposomes feature a central aqueous cavity suitable for entrapping hydrophilic ingredients. At the same time, hydrophobic components can be encapsulated within the nanoliposome membrane, composed of either a single bilayer or a double layer of phospholipids [280]. One study aimed to enhance the durability of ACNs from red cabbage within the gastrointestinal system by incorporating red cabbage extract (RCE) into solid lipid nanoparticles (SLNs) using a water-in-oil microemulsion. The resilience of RCE-SLNs was found to be stronger at pH 3.0 (gastric fluid) compared to pH 5.0 (intestinal fluid); storing them at 25 °C preserved the ACNs, ensuring increased absorption and potential health benefits [281]. Similarly, a stable mangosteen peel extract (MPE) nanoemulsion was achieved through high-speed homogenization with specific ratios of virgin coconut oil, Tween 80, and Span 80. This resulting MPE-NE remained physically stable for 28 days and exhibited a shelf life of up to one year, suggesting its potential for topical applications [282]. Furthermore, nanoliposomes incorporating pistachio green hull extract (PHE-NL) and soy lecithin (1–3%) demonstrated improved preservation of ACNs and lipid oxidation stability compared to PHE or empty nanoliposomes, attributed to the presence of polyphenols [283].

## 8. Conclusions and Perspectives

Exploring ACNs as immunomodulatory dietary supplements presents a promising avenue for harnessing the potential of natural compounds to support and enhance immune system function. The extensive research discussed in this review highlights the multifaceted mechanisms through which ACNs exert their immunomodulatory effects, ranging from antioxidant and anti-inflammatory activities to direct interactions with immune cells. The diverse sources of ACNs, including various fruits, vegetables, and plants, offer various options for individuals seeking to incorporate these compounds into their diets. However, the full realization of the immunomodulatory benefits of ACNs hinges upon overcoming the challenges associated with their limited bioavailability. Nanostrategies have emerged as innovative solutions for enhancing the absorption and bioavailability of ACNs, thereby maximizing their therapeutic potential. Nanocarriers and encapsulation techniques hold the promise of optimizing the delivery of ACNs to target tissues and cells, ultimately amplifying their immunomodulatory effects.

Future research should focus on establishing a comprehensive understanding of the molecular mechanisms underlying anthocyanin-immune cell interactions, unraveling their intricate signaling pathways, and deciphering their precise impact on immune system regulation. Additionally, continued efforts in developing advanced nanoformulations are paramount to ensure the effective delivery and enhanced bioavailability of ACNs. As personalized nutrition gains prominence, tailoring anthocyanin-based interventions to specific populations and immune-related conditions could provide valuable insights into their clinical applications. Collaborative endeavors between nutritionists, immunologists, pharmacologists, chemists, and nanotechnologists are essential to translate the potential demonstrated in preclinical and early clinical studies into tangible health benefits for individuals of all ages. Continued interdisciplinary research and translational efforts will pave the way for a future where ACNs contribute significantly to optimizing immune system function and promoting overall health.

## Figures and Tables

**Table 1:** Immunomodulatory potential of anthocyanins, their aglycons, and metabolites.

Anthocyanin and Its Aglycons	Dose	Methodology	Major Findings	Reference
Cyanidin-3-glucoside	100 μM	In Vitro Osteoclasts from C57BL/6J mice cultured with M-CSF (30 ng/mL) and the receptor activator of a nuclear factor kappa B ligand (RANKL) (100 ng/mL) and supplemented with the test sample.	Inhibited osteoclast differentiation and formation induced by RANKL.	[60]
Protocatechuic acid (anthocyanin metabolite)	0–25 μM	In Vitro Osteoclasts from ICR mice were cultured in the presence of M-CSF (30 ng/mL) and RANKL (50 ng/mL) and treated with the test sample.	Inhibited RANKL-induced osteoclast differentiation and suppressed the bone-resorbing activity of mature osteoclasts.	[61]
	25 mg/kg/d	In Vivo Nine days of administration in LPS (5 mg/kg)-induced bone destruction in male ICR mice.	Recovered the bone volume per tissue volume, trabecular separation, and trabecular number.	
Delphinidin	50 μM	In Vitro Jurkat E6-1 cells were preincubated with gadolinium and *N*-(4-[3,5-bis(trifluoromethyl)-1H-pyrazol-1-yl]phenyl)-4-methyl-1,2,3-thiadiazole-5-carboxamide for 15 min.	Induced Ca^2+^ and Sr^2+^ influx through the CRAC channel and induced NFAT pathway activation; thereby, the production of IL-2 occurs.	[62]
Anthocyanidins	5–20 μM	In Vitro TPA (20 ng/mL)-induced cell transformation was investigated in the parental JB6 Cl41 cells or AP-1-luciferase stable transfectant JB6 cells.	Ortho-dihydroxyphenyl structure (delphinidin) on the B-ring of aglycon inhibited cell transformation and activator protein-1 transactivation.	[63]
Delphinidin	25–100 μM	In Vitro RAW264 cells were treated with LPS (40 ng/mL) for 6 h.	Inhibited MAPK-mediated COX-2 expression.	[64]
Cyanidin-3-*O*-galactoside and cyaniding-3-sambubioside rich extract of *Sambucus ebulus*	200 mL infusion	Interventional study Fifty-three volunteers enrolled in a 4-week intervention involving the consumption of infusion.	Decreased pro-inflammatory status and complemented activity markers in healthy volunteers.	[65]
Cyanidin-3-*O*-glucoside and peonidin-3-*O*-glucoside rich fraction of black rice germ and bran.	0–200 μg/mL	In Vitro A549 cells and THP-1 macrophages exposed to SARS-CoV-2 spike glycoprotein S1 (100 ng/mL).	Inhibited NF-κB activity and the NLRP3 inflammasome pathway, leading to the inhibition of inflammatory genes and cytokine secretions.	[66]
Cyanidin-3-*O*-glucoside	0–20 μg/mL			
Peonidin-3-*O*-glucoside	0–20 μg/mL			
Cyanidin-3-*O*-glucoside delivered by enteric sodium alginate	25 mg/kg/d	In Vivo Male BALB/c mice received two sensitization doses of 100 µg ovalbumin and 2.0 mg alum, administered in 200 µL of phosphate-buffered saline via intraperitoneal injection on day 0 and day 14.	Enhanced the intestinal epithelial barrier by up-regulating the tight junction protein expression and promoting secretory IgA and β-defensin secretion and regulated Th1/Th2 immune balance in the intestinal mucosa.	[67]
Anthocyanin-rich roselle extract	0–400 mg/kg	In Vivo 1-day-old Ross 308 broiler chicks were fed on basal diets supplemented with extract for 35 days.	Improved metabolic functions, blood biochemistry, intestinal morphology, antioxidant activity, immune status, and higher ω-3 content in the breast muscle.	[68]
Cyanidin-3-glucoside-enriched diet.	26 mg/kg/d	Ex Vivo Myocardial ischemia/reperfusion (I/R) injury in mice. Pretreated with the sample for a month.	Diet changed the microbiome and the transplantation of the fecal microbiota transferred the cardioprotection.	[69]
Total anthocyanin from boysenberry and apple juice concentrate	2.5 mg/kg	In Vivo Ovalbumin-induced airway inflammation in male C57BL/6J mice.	Reduced immune cell infiltration and tissue damage.	[71]
Cyanidin 3-*O*-glucoside	10 mg/kg	In Vivo Five-week-old male spontaneously hypertensive and Wistar-Kyoto rats were treated daily for a period of 15 weeks.	Treatment normalized the splenic production of TNFα and IFNγ and the proportion of CD62L^lo^ and CD62L^−^ helper T-cells.	[74]
Cyanidin-3-*O*-glucoside	25 mg/kg	In Vivo Treated twice per week for six consecutive administrations in Sprague Dawley with bovine type II collagen-induced arthritis.	The study suggests that CD38^+^ NK cells inhibiting Treg cell differentiation may contribute to the immune imbalance seen in both rheumatoid arthritis and collagen-induced arthritis.	[75]
	25–100 μM	In Vitro RA synovial fibroblasts collected from arthritic patients.		
	50 μM	In Vitro Mononuclear cells were collected from arthritic patients.		
	50 μM	In Vitro Mononuclear cells were cocultured with CD38^+^ NK cells.		
Delphinidin-3-*O*-glucoside and cyanidin-3-*O*-glucoside	50–600 µg/mL	In Vitro Both the compounds alone or in combinations were added and incubated with HCT-116 and HT-29 human colorectal cancer cells for 24 h.	Both showed potential for binding with and inhibiting immune checkpoints, PD-1 and PD-L1, which can activate the immune response in the tumor microenvironment and induce cancer cell death.	[76]
Anthocyanin-rich mix	40 mg/kg BW	In Vivo High-fat-diet-induced obesity, dyslipidemia, insulin resistance, and steatosis in C57BL/6J mice.	Increased glucagon-like peptide-2 levels, the intestinal hormone that upregulates tight junction protein expression.	[77]
Anthocyanin-rich mix (AC mix)	5 μg/mL	In Vitro Caco-2 cell monolayers were pre-incubated for 24 h with IFNγ (10 ng/mL) to upregulate the TNFα receptor.	PCA, C3G, and the AC mix, fully prevented p65, ERK1/2, and MLC phosphorylation. D3G inhibited TNFα-triggered p65 phosphorylation; Peo3G had no effect.	
Protocatechuic acid (PCA)	0.5 μM			
Delphinidin-3-*O*-glucoside (D3G)	0.5 μM			
Cyanidin-3-*O*-glucoside (C3G)	1 μM			
Peonidin-3-*O*-glucoside (Peo3G)	0.1 μM			
Cyanidin-3-glucoside-rich anthocyanin extract of *Lonicera caerulea* fruit	0–0.8 mg/mL	In Vitro Human hepatoma cell line SMMC-7721.	Inhibits proliferation of the tumor cells.	[78]
	50, 100 and 200 mg/kg BW	In Vivo Tumor-bearing male Kunming strain mice model was established using the murine H22 hepatoma cells line. Treated with sample for 15 days.	Extract effectively combats tumours by dynamically adjusting the redox balance and enhancing immunoregulatory activity.	
Cyanidin-3-*O*-glucoside	20 and 40 μM	In Vitro TNF-α-stimulated intestinal cells tested on endothelial cell activation by using cocultures (Caco-2, HUVEC, and mononuclear cells)	Inhibited NF-κB pathway in epithelial cells.	[79]
Cyanidin chloride and cyanidin 3-*O*-glucoside	0–25 μM	In Vitro LPS- (100 ng/mL) or IFNγ- (10 ng/mL) induction in immortalized mouse microglial (bv-2) cells pretreated with samples.	Both compounds reduced ROS production in a dose-dependent manner. Cyanidin chloride showed a maximum decrease in NO production.	[80]
Cyanidin-3-glucoside chloride	1.25–5.0 μg/mL	In Vitro EL-4 T cells were pretreated with various concentrations of the sample for 1 h and stimulated with PMA/ionomycin for 16 h.	Suppressed Th2 activation through the downregulation of Th2 cytokines and the GATA3 transcription factor.	[81]
Black raspberry Ethanol extract	0–200 μg/mL	In Vitro Normal donor peripheral blood mononuclear cells were induced with IL-6 and GM-CSF (10 ng/mL) and were pretreated with samples for 7 days.	Limited expansion of myeloid-derived suppressor cells (MDSC). Attenuated IL-6-mediated phosphorylation of STAT3 and IL-2-induced STAT5 phosphorylation. Pretreatment of immune cells inhibited MDSC expansion and IL-6-mediated STAT3 signalling.	[82]
Cyanidin-3-rutinoside	0–200 μM			
Delphinidin-3-glucoside	0–100 μM	In Vitro BM185 cells were incubated with test samples for 48 h.	Inhibited cell proliferation dose-dependently.	[83]
Cyanidin-3-rutinoside	0–100 μM			
Anthocyanin-rich berry extract	10–100 μg/mL			
Cyanidin-3-rutinoside	50 mg/kg/d	In Vivo Eight-week-old female Balb/c mice challenged with BM185 cells treated with samples for 21 days.	Moderate induction of caspases.	[83]
Anthocyanin-rich berry extract	500 mg/kg/d			
Cyanidin glycosides-rich juice (total polyphenol = 236 mg)	330 mL/d	*Clinical trial* Randomized, crossover study divided into five periods each lasting two weeks.	Enhanced antioxidant status, reduced oxidative DNA damage, and stimulated immune cell functions (IL-2 production).	[84]
Proanthocyanidins	30 µM	In Vitro LPS (10 ng/mL) or IL-4 (1 ng/mL) activated murine J774 macrophages. LPS (10 ng/mL) or IL-4 (1 ng/mL) activated human U-937 monocytes.	Enhanced the expression of Arg-1 and MRC-1 in a dose-dependent manner. Enhanced the phosphorylation of STAT6. Increased CCL-17 expression.	[85]
Delphinidin 3-*O*-β-d-glycoside	50 µM	In Vitro LPS-induced mesenchymal stem cells.	Modified the immunomodulatory properties and increased the IL-10 production.	[86]
Black lentil water extract	600 mg/kg BW	In Vivo Azoxymethane/dextran sodium sulphate-induced C57BL/6 mice model.	Modulated cytokines are involved in mediating communication between immune cells and anti-inflammatory action. Reduced colon mucosa of markers like IL-1β and IL-6 and gene expression, such as CXCR2 and CSF3.	[87]
Delphinidin 3-*O*-(2-*O*-*β*-D-glucopyranosyl-*α*-L-arabinopyranoside rich lentil extract	41 mg/kg BW			
Delphinidin chloride	20 and 40 µM	In Vitro CD4^+^ T cells were purified from the spleen of C57BL/6 mice and were activated with 1 µg/mL of plate-bound anti-CD3 and plate-bound anti-CD28 antibodies in the presence samples for 3 days.	Promoted the differentiation of Tregs and enhanced immune response control.	[88]
	1.5 and 3 mg/kg	In Vivo *allograft model* Five-week-old male C57BL/6 mice were subcutaneously injected with a fixed number of P815 cells	Reduced activated T cells by promoting Treg differentiation.	
Delphinidin	10^−2^ g/L	In Vitro T-lymphocytes from non-metabolic-syndrome and metabolic syndrome patients were stimulated for 24 h with the sample and 5 µg/mL of PHA.	Inhibited Ca^2+^ signalling via reduced store-operated Ca^2+^ entry and release and the subsequent decrease of HDAC and NFAT activations. Also inhibited ERK1/2 activation and suppressed differentiation of T cells toward Th1, Th17, and Treg without affecting Th2 subsets. Prevented the PHA-induced proliferation in cells isolated from WT. Inhibited HDAC activity by a mechanism downstream of its effect on Ca^2+^ signaling.	[89]
		Peripheral blood mononuclear cells isolated either from 57BL/6 females ERα Wild-Type or Knock-Out mice stimulated for 48 h with 5 µg/mL of PHA.		
		T-lymphocytes stimulated with thapsigargin (1 µM).		
Delphinidin	0–20 µM	In Vitro Normal Human epidermal keratinocytes treated with rhIL-22 and TPA.	Suppressed key kinases involved in psoriasis pathogenesis and alleviated IMQ-induced murine psoriasis-like disease, suggesting a novel PI3K/AKT/mTOR pathway modulator for psoriasis.	[90]
Delphinidin	0–50 µM	In Vitro Caspase activation in human prostate LNCaP and Du145 cells treated with sample for 12 h.	Sensitized prostate cancer cells to TRAIL-induced apoptosis by inducing DR5, causing caspase-mediated HDAC3 cleavage.	[91]
Delphinidin	0–50 µM	In Vitro MH7A and Jurkat cells were treated with the sample and cultured for 2 h with TNFα or LPS.	Inhibited NF-ĸB expression, thereby decreasing cytokine expression.	[92]
Resveratrol and peonidin 3-*O*-glucoside rich red grape vine leaf extract	100 μg/mL	In Vitro THP-1 cells were stimulated with 2 μg/mL Cy3-labeled DNA for 4 h.	Restricted the AIM2 inflammasome activation by preventing DNA entry.	[93]
	140 mg/kg	In Vivo Treated in IMQ-induced BALB/c mice (6–9 weeks old) model for 5 days.	Inhibited proinflammatory caspase-1 activation, IL-1β maturation, and IL-17 production.	
Malvidin-3-*O*-glucoside	10 µM	In Vitro Murine microglia primary cultures primed and activated with LPS.	Targets NLRP3, NLRC4, and AIM2 inflammasomes, subsequently reducing caspase-1 and IL-1β protein levels. Stopped the release of IL-1β in the hippocampus.	[94]
	12.5 mg/kg/d	In Vivo Chronic unpredictable stress was subjected to the mice for 28 days, consisting of different stressors. A 2-week pretreatment was followed.		
Malvidin-3-*O*-glucoside	0.5 µg/kg/d	In Vivo C57BL/6 male mice were treated with the sample for 2 weeks before and throughout repeated social defeat stress and then performed social avoidance/interaction testing.	Modulated synaptic plasticity by increasing histone acetylation of the regulatory sequences of the Rac1 gene.	[95]

## Data Availability

Not applicable.
